# Postoperative Serum Quinolinic Acid and 3-Hydroxykynurenine in Dogs with Congenital Portosystemic Shunt: A Pilot Study of Their Association with Postattenuation Neurologic Signs

**DOI:** 10.3390/vetsci13040308

**Published:** 2026-03-24

**Authors:** Shoma Mikawa, Yuto Ishimaru, Yasuhiko Okamura

**Affiliations:** 1 Faculty of Veterinary Medicine, Okayama University of Science, Imabari 794-8555, Ehime, Japan; y-okamura@ous.ac.jp; 2 Seiwadai Animal Hospital, Kawanishi 666-0142, Hyogo, Japan; y.i.0509.140@gmail.com

**Keywords:** congenital disorder, kynurenine pathway, postattenuation seizures, surgical ligation, tryptophan metabolism

## Abstract

Postattenuation neurologic signs (PANS) are serious complications following surgical ligation of congenital portosystemic shunts (cPSS) in dogs, but their pathogenesis remains incompletely understood. We hypothesized that surgical ligation alters hepatic tryptophan metabolism through increased substrate delivery and inflammation, leading to changes in kynurenine pathway metabolites that may contribute to PANS. Ten dogs diagnosed with cPSS between April 2019 and December 2023 underwent surgical ligation. Serum samples were collected preoperatively and on postoperative days 1, 2, and 3. Serum concentrations of quinolinic acid (QA) and 3-hydroxykynurenine (3OHKYN) were measured using ELISA in this retrospective observational study. Dogs exhibiting neurological signs within 3 days postoperatively were classified as having PANS. Serum QA concentrations showed no significant postoperative changes and did not differ between dogs with and without PANS. In contrast, serum 3OHKYN concentrations decreased significantly after surgery (*p* = 0.002). Dogs that developed PANS had significantly lower serum 3OHKYN concentrations on postoperative day 1 (*p* = 0.048) and exhibited greater reductions from baseline on postoperative days 1 and 3 (*p* = 0.024 and 0.017, respectively). These preliminary findings suggest that postoperative reductions in 3OHKYN, rather than increases in QA, are associated with PANS development, indicating that altered tryptophan metabolism may play a role in its pathogenesis and warrant further investigation in larger studies.

## Simple Summary

Some dogs are born with an abnormal blood vessel that allows blood to bypass the liver. Surgery to close this vessel can be curative, but some dogs develop serious nervous system problems soon after the operation, such as abnormal behavior, tremors, or seizures. The cause of these postoperative neurologic signs is still unclear. In this preliminary study, we investigated whether surgery changes the breakdown of the amino acid tryptophan in the body, which may influence brain function. We analyzed blood samples from ten dogs before surgery and on days 1, 2, and 3 after surgery. We measured two substances related to tryptophan metabolism: quinolinic acid, which may overstimulate the brain, and 3-hydroxykynurenine, another metabolic product. Quinolinic acid did not change after surgery and was not linked to postoperative neurologic signs. However, 3-hydroxykynurenine decreased after surgery, and dogs that developed neurologic signs showed larger decreases, especially on the first day after surgery. These preliminary findings suggest that changes in tryptophan metabolism after surgery may contribute to postoperative neurologic signs and could help guide future prevention strategies.

## 1. Introduction

Congenital portosystemic shunt (cPSS) is a common congenital disorder in dogs. It is characterized by an abnormal vessel connecting the portal vein to the systemic circulation, causing portal blood to bypass the liver. This reduces hepatic blood flow, leading to microhepatia and impaired liver function. Ammonia, normally detoxified by the liver, accumulates in the systemic circulation, contributing to ammonium urate stone formation and hepatic encephalopathy [1,2]. Depending on the type of shunt, cPSS can potentially be cured through surgical intervention. Surgical ligation of the shunting vessel is the definitive treatment, as it can restore liver function and reduce hyperammonemia [3,4]. However, complications such as postattenuation neurological signs (PANS) and postattenuation seizures are well-documented risks of this procedure [4,5]. The PANS, characterized by symptoms such as depression, ataxia, tremors, loss of vision, and seizures, occurs between surgery and discharge, and can be fatal. The incidence of PANS is 1.6–27.3%, with varying survival rates (PANS: 50% [0–100%], postattenuation seizures: 37.5% [0–75%]). Risk factors for PANS include preoperative hepatic encephalopathy, advanced age, extrahepatic portosystemic shunt morphology, and breed type; however, the mechanisms remain unclear [5]. Similarly, elevated endogenous benzodiazepine ligands in dogs with cPSS suggest that endogenous benzodiazepine receptor stimulation and withdrawal may trigger seizures [6]. However, studies in this field remain limited.

**Figure 1:**
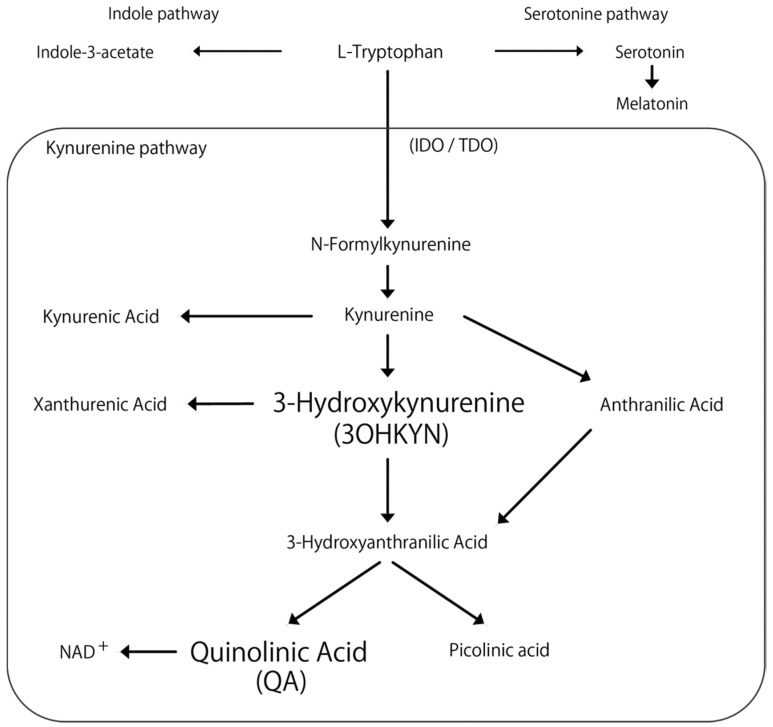
Flowchart of the kynurenine pathway. The arrows indicate metabolic changes. IOD: indoleamine 2,3-dioxygenase. TDO: tryptophan 2,3-dioxygenase. NAD+: nicotinamide adenine dinucleotide.

Tryptophan, an essential amino acid, produces various metabolites with important physiological activities and has been associated with neurological and liver diseases [7,8]. Primary metabolic pathways include the kynurenine, serotonin, and indole pathways (Figure 1). In humans, the majority of tryptophan is metabolized through the kynurenine pathway [9,10], with its metabolites linked to conditions such as schizophrenia, depression, Alzheimer’s, and Parkinson’s diseases [11,12,13,14].

Quinolinic acid (QA) and 3-hydroxykynurenine (3OHKYN) are known for their convulsant properties. The QA induces seizures in mice [15,16], and 3OHKYN, which elevates brain QA concentrations, reportedly increases in children with seizures [17]. Notably, the enzymes indoleamine 2,3-dioxygenase 2 and tryptophan 2,3-dioxygenase, which regulate this pathway, are mainly expressed in the liver [18]. However, only one study has examined kynurenine pathway metabolites in dogs with cPSS, and the study reported increased tryptophan, glutamine, 5-hydroxyindoleacetic acid, and QA concentrations in the cerebrospinal fluid (CSF) of dogs with cPSS compared to healthy dogs [19]. These findings suggest abnormal tryptophan metabolism in dogs with cPSS, potentially due to changes in hemodynamics and hepatic metabolic function following surgical intervention. Inflammation is one of several factors that can activate the kynurenine pathway, implying that surgical trauma may alter metabolite concentrations [20]. Further reports also show that blood tryptophan levels did not change 3 months after surgery in dogs with cPSS [21]; however, no studies have investigated tryptophan metabolites in the perioperative period.

We hypothesized that surgical ligation would increase tryptophan delivery to the liver and that inflammation would exacerbate kynurenine pathway metabolism, increasing QA and 3OHKYN concentrations, and possibly contributing to PANS. Accordingly, this study aimed to measure serum QA and 3OHKYN concentrations before and after surgery in cPSS dogs and compare them between dogs with and without PANS.

## 2. Materials and Methods

This retrospective study included dogs that underwent surgical ligation of a shunting vessel after being diagnosed with a cPSS by contrast-enhanced computed tomography at the Veterinary Teaching Hospital of Okayama University of Science between April 2019 and December 2023. The dogs that underwent shunt vessel ligation in two separate procedures were excluded. Serum samples were obtained from leftover specimens used in clinical tests conducted preoperatively and on postoperative days 1, 2, and 3. All blood samples were collected before feeding. The samples were centrifuged immediately after collection, and after the required tests were performed, they were promptly stored at −80 °C until analysis. Dogs that exhibited neurological symptoms such as seizures, disorientation, or compulsive swimming movements within 3 days postoperatively were assigned to the PANS group. The dogs were continuously monitored for 72 h postoperatively, and the presence or absence of neurological signs was recorded.

Serum QA and 3OHKYN concentrations were measured using the enzyme-linked immunosorbent assay (ELISA). Serum samples, diluted in 10- and 2-fold, were analyzed using the Universal Quinolinic Acid ELISA Kit (FY-EU12360, Wuhan Feiyue Biotechnology, Wuhan, China) and Canine 3-Hydroxykynurenine ELISA Kit (MBS7277704, MyBioSource, Inc., San Diego, CA, USA), respectively. The reactions were performed in duplicate, and absorbance at 450 nm was measured using a microplate reader (SH-100Lab, Corona Electric Co., Ltd., Ibaraki, Japan). Serum C-reactive protein (CRP) concentrations were measured using a laser nephelometric immunoassay with a Laser CRP-2 analyzer (Arrows Co., Ltd., Osaka, Japan) according to the manufacturer’s instructions.

Data are presented as the mean ± standard deviation for normally distributed variables and as median (minimum–maximum) for non-normally distributed variables. Normality tests were performed using the Shapiro–Wilk normality test. Repeated measures analysis of variance for normally distributed data or the Friedman test for non-normal distributions was used to test differences in the temporal changes in blood concentrations, and Bonferroni multiple comparisons were used for intergroup comparisons. Continuous data with normal distributions were compared between groups according to the presence or absence of the binary variable using Welch’s *t*-test, non-normally distributed continuous data were analyzed using the Mann–Whitney U test, and categorical data were compared using Fisher’s exact test. Correlations between continuous variables were assessed using Pearson’s correlation coefficient for normally distributed data and Spearman’s correlation coefficient for non-normally distributed data. Statistical significance was set at *p* < 0.05. Data were analyzed using EZR on R Commander (ver. 1.51; Saitama Medical Center, Jichi Medical University, Shimotsuke, Japan) [22].

## 3. Results

### 3.1. Study Population

During the study period, 15 dogs were diagnosed with cPSS and underwent surgical ligation of the shunting vessel. Of these, five dogs were excluded from the study because complete ligation could not be achieved or was performed through multiple surgeries. The remaining 10 dogs with cPSS (seven males [six neutered males] and three females [none spayed]) were included in this study, with a median age of 23 months (range: 8–82 months), and the mean body weight of 4.70 ± 2.37 kg. Blood samples were collected from all dogs on the day before surgery and on postoperative day 3. However, samples were also available for nine dogs on postoperative day 1 and for eight dogs on postoperative day 2; the unavailable samples were from dogs in the non-PANS group. Full ligation of the shunt vessel was performed in nine dogs, whereas one dog in the PANS group underwent partial ligation due to portal hypertension.

Three dogs developed PANS postoperatively. In the PANS group, the median age was 50 ± 30 months, and the mean body weight was 5.59 ± 2.44 kg. One dog showed transient ataxia and incoordination on day 3 postoperatively but recovered thereafter. Another dog exhibited decreased consciousness and seizures on day 2 postoperatively and died on day 4 postoperatively. The third dog had decreased consciousness and seizures on day 3 postoperatively but was discharged on day 21 postoperatively following intensive care. All three dogs had normal blood ammonia levels (16.0–75.0 μg/dL) when they developed PANS, so hepatic encephalopathy was not considered to be the cause. Preoperatively, two of the three dogs received oral levetiracetam prophylactically. In the non-PANS group, the mean age was 21 ± 9 months, and the mean body weight was 4.32 ± 2.42 kg. No neurological symptoms were observed in the non-PANS group. Postoperative ammonia levels were within the normal range.

Preoperatively, five of the seven dogs received prophylactic oral levetiracetam (Supplementary Table S1). Only two dogs in the PANS group had a history of hepatic encephalopathy, and their condition was controlled at the time of surgery. There were no significant differences in age, body weight, sex, or shunt morphology between the PANS and non-PANS groups. Furthermore, there were no significant differences between the two groups regarding the presence of hepatic encephalopathy, preoperative administration of levetiracetam, or feeding of a hepatic therapeutic diet.

### 3.2. Serum Quinolinic Acid and 3-Hydroxykynurenine

**Figure 2:**
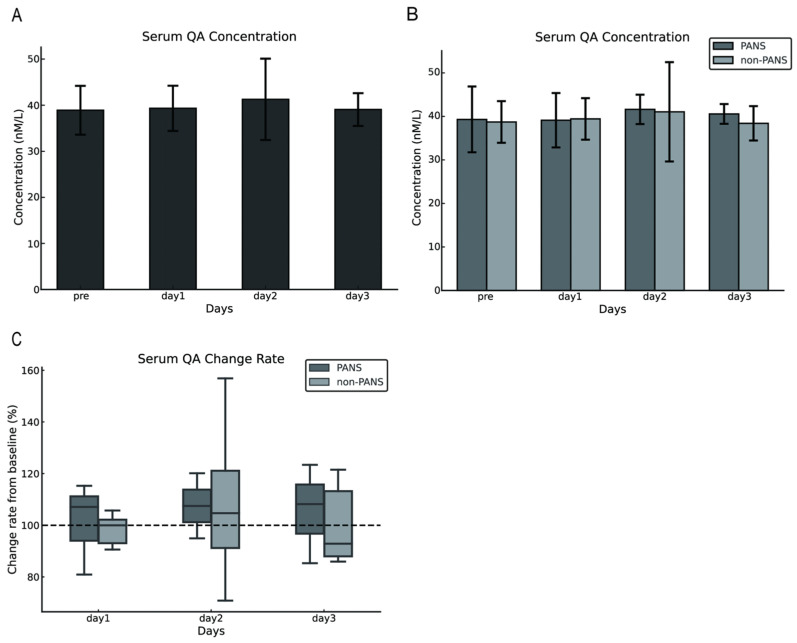
Changes in serum QA concentrations following shunt vessel ligation in dogs with cPSS. No change was observed in serum QA concentrations before and after surgery (**A**). No difference was observed in changes in serum QA concentrations between the PANS and non-PANS groups (**B**,**C**). The error bars represent either the standard deviation or the minimum and maximum values. The dashed line in Figure C represents the baseline set at 100% for each individual case (with the pre value defined as 100%). QA, quinolinic acid; cPSS, congenital portosystemic shunts; PANS, postattenuation neurologic signs.

The serum QA concentrations in dogs with cPSS were 38.90 ± 5.3 nmol/L preoperatively and 39.32 ± 4.91, 41.27 ± 8.83 nmol/L, and 39.06 ± 3.56 nmol/L on postoperative days 1, 2, and 3, respectively. No difference was observed between preoperative and postoperative serum QA concentrations (*p* = 0.821; Figure 2A). Similarly, comparisons between the PANS and the non-PANS groups indicated no statistical differences in serum QA concentrations (Figure 2B). The rate of change from baseline (preoperative values) was calculated, and no statistical differences were observed between the PANS and non-PANS groups (Figure 2C).

**Figure 3:**
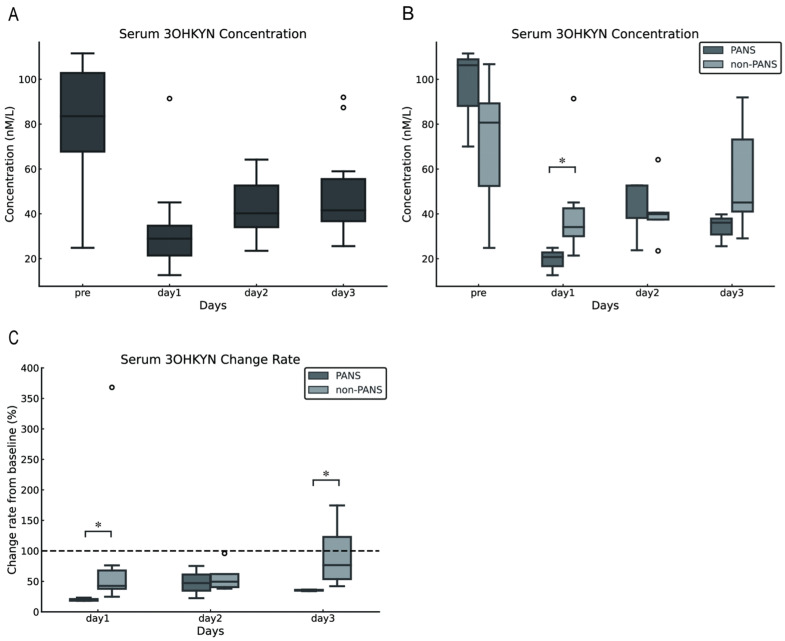
Changes in serum 3OHKYN concentrations following shunt vessel ligation in dogs with cPSS. Serum 3OHKYN concentrations tended to decrease after surgery (**A**). On postoperative day 1, serum 3OHKYN concentrations were significantly lower in the PANS group compared with the non-PANS group (**B**). When the preoperative level was set as 100%, serum 3OHKYN concentrations in the PANS group significantly decreased on postoperative days 1 and 3 (**C**). The error bars represent the minimum and maximum values. The white circles indicate outliers. Asterisks denote *p* < 0.05. The dashed line in Figure C represents the baseline set at 100% for each individual case (with the pre value defined as 100%). 3OHKYN, 3-hydroxykynurenine; cPSS, congenital portosystemic shunts; PANS, postattenuation neurologic signs.

The serum 3OHKYN concentrations in dogs with cPSS were 83.50 (24.83–111.55) nmol/L preoperatively and 28.89 (12.65–91.38), 40.22 (23.51–64.16), and 49.59 (25.60–91.94) nmol/L on postoperative days 1,2, and 3, respectively. Compared with preoperative levels, serum 3OHKYN concentrations significantly decreased postoperatively (*p* = 0.002). However, multiple comparisons against preoperative values revealed no statistically significant differences (*p* = 0.094 for each comparison; Figure 3A). Furthermore, the serum 3OHKYN concentrations in the PANS group were significantly lower than those in the non-PANS group on postoperative day 1 (*p* = 0.048). However, no significant differences were observed preoperatively and on postoperative days 2 and 3 (*p* = 0.786, 0.117, respectively; Figure 3B). The rate of change from baseline (preoperative values) was calculated, and significant differences were found between the PANS and non-PANS groups on postoperative days 1 and 3 (*p* = 0.024 and 0.017, respectively; Figure 3C).

Serum CRP concentrations increased postoperatively and showed a decreasing trend over time; however, no significant differences were observed among the time points. Furthermore, there were no significant differences in CRP concentrations between the PANS and non-PANS groups (Supplementary Figure S1).

No differences in preoperative serum concentrations of QA and 3OHKYN were observed with respect to sex, shunt morphology, preoperative administration of levetiracetam, use of a hepatic therapeutic diet, or presence of concurrent surgery. Furthermore, no correlations were found between preoperative serum QA or 3OHKYN concentrations and age or body weight. No correlations between serum QA and 3OHKYN concentrations were observed either preoperatively or postoperatively. Serum CRP concentrations showed a significant positive correlation with serum 3OHKYN on postoperative day 2; however, no significant correlations were observed with QA or 3OHKYN at the other time points (Supplementary Figure S2).

## 4. Discussion

In this study, we evaluated the preoperative and postoperative serum QA and 3OHKYN concentrations in dogs with cPSS. To date, only one study has evaluated QA concentrations in the CSF and serum of dogs with cPSS [19], and to our knowledge, no study has assessed serum 3OHKYN concentrations. Furthermore, no previous research has evaluated kynurenine pathway metabolites following shunt vessel ligation surgery, making this study the first report on this topic.

A study evaluating the blood amino acid profile 3 months after surgery in dogs with cPSS reported no change in postoperative blood tryptophan concentrations [21]; however, no research has examined changes occurring immediately after surgery that could potentially lead to PANS. Moreover, the study by Devriendt et al. (2021) employed a gradual surgical attenuation [21], whereas our study included only dogs that underwent complete ligation, which may result in different postoperative tryptophan dynamics. We hypothesized that shunt vessel ligation would increase tryptophan inflow to the liver via the portal vein, and that inflammation caused by surgical stress would enhance kynurenine pathway metabolism, leading to an increase in its metabolite QA, which could contribute to the development of PANS. However, in this study, no changes in serum QA concentrations were observed between the preoperative and postoperative periods. Previous research reported substantially higher QA concentrations in the CSF of dogs with cPSS compared to healthy dogs, whereas serum QA concentrations were markedly lower [19]. Limited permeability of QA is exhibited across the blood–brain barrier [23]. Similarly, in the current study, a discrepancy between QA concentrations in the serum and CSF may have been present.

Next, we investigated the blood concentration of 3OHKYN, a metabolic precursor of QA that crosses the blood–brain barrier more easily [23,24]. We expected that kynurenine metabolites would increase following shunt vessel ligation; However, serum 3OHKYN concentrations decreased. Furthermore, the serum 3OHKYN concentration in the PANS group was substantially lower than that in the non-PANS group on postoperative day 1. When expressed as the rate of change relative to preoperative levels, 3OHKYN was also substantially reduced in the PANS group on postoperative day 3. These findings suggest that a greater reduction in serum 3OHKYN concentrations may be associated with the development of PANS. The 3OHKYN is a seizure-inducing substance, and elevated levels in the CSF have been reported in infantile seizures [17]. Given its ability to cross the blood–brain barrier [23], serum 3OHKYN concentration in this study was considered to partially reflect the concentration in the CSF. The fact that the results in this study were contrary to expectations is highly intriguing.

In addition to QA and 3OHKYN, other metabolites of the kynurenine pathway include kynurenic acid, picolinic acid, and xanthurenic acid. Whereas QA and 3OHKYN are known as neurotoxic metabolites, kynurenic acid and picolinic acid are neuroprotective metabolites. The balance among these metabolites may play a role in neurological diseases [25]. Although xanthurenic acid has not been linked to neurological disorders, it binds to glutamate receptors [26,27]. Excessive activation of glutamate receptors can lead to excitotoxicity, which can cause neurological symptoms, and thus, the possibility of its involvement cannot be ruled out. The observed decrease in 3OHKYN in this study may suggest increased metabolism into these other non-QA metabolites of the kynurenine pathway. Furthermore, tryptophan metabolism also involves the serotonin and indole pathways, in addition to the kynurenine pathway [28]. Increased metabolism through these alternative pathways may partly explain the decrease in 3OHKYN observed in the present study. Serotonin functions as an autacoid and neurotransmitter in the brain and is associated with seizure activity [29,30]. Therefore, future research, including the metabolomic analysis of the entire tryptophan metabolic network beyond the kynurenine pathway, may be useful in elucidating the mechanisms underlying the development of PANS.

This study had some limitations. First, we were unable to examine the baseline serum concentrations of QA and 3OHKYN in healthy dogs. Although one previous study reported that serum QA concentrations were lower in dogs with cPSS than in healthy controls [19], available data on serum QA concentrations in dogs remain very limited. In contrast, to the best of our knowledge, no previous studies have evaluated serum 3OHKYN concentrations in dogs. Therefore, it remains unclear whether the concentrations of these metabolites were already abnormal in dogs with cPSS before surgery. Second, we could not evaluate QA and 3OHKYN concentrations in the CSF. Although this study unexpectedly showed no change in serum QA concentrations postoperatively and a decrease in serum 3OHKYN concentrations, how the concentrations of these metabolites actually change within the brain remains unclear. Given that 3OHKYN can cross the blood–brain barrier, its blood concentration is considered to partially reflect its concentration in the brain; however, measurement of kynurenine pathway metabolites in the cerebrospinal fluid is necessary to assess their association with the development of PANS. Third, this study did not include a control group of dogs undergoing other types of abdominal surgery. Therefore, it remains unclear whether the observed postoperative decrease in serum 3OHKYN is specific to shunt ligation or represents a more general response to surgical trauma or systemic inflammation associated with major abdominal surgery. In the present study, only serum 3OHKYN on postoperative day 2 showed a significant correlation with CRP, whereas no correlations were observed at the other time points. It remains unclear whether this finding was incidental or reflects systemic inflammation. We were also unable to fully evaluate the effects of surgical trauma–induced inflammation on the kynurenine pathway. Some animals included in this study underwent concurrent surgeries, such as cystotomy or cholecystectomy. However, no differences in serum QA, 3OHKYN, or CRP concentrations were observed between dogs with and without concurrent surgery. Future studies including dogs undergoing non-shunt abdominal procedures would help clarify the extent to which surgical trauma influences kynurenine pathway metabolites. Fourth, only three dogs that developed PANS were included in this study. This small sample size substantially limits the statistical power of the analyses and the generalizability of the findings, particularly for comparisons between dogs with and without PANS. Therefore, the present results should be interpreted as preliminary observations and require confirmation in larger cohorts. In addition, the use of medications such as levetiracetam may have influenced kynurenine pathway metabolism and neurological outcomes, and this potential confounding effect could not be fully controlled in the present study. Finally, because this was an observational study, the association between postoperative changes in 3OHKYN and the occurrence of PANS does not imply a causal relationship. It remains unclear whether the decrease in 3OHKYN contributes to the development of PANS or whether the occurrence of PANS, or other underlying postoperative processes, leads to alterations in kynurenine pathway metabolites.

## 5. Conclusions

We found that while serum QA concentrations did not change following shunt vessel ligation in dogs with cPSS, serum 3OHKYN concentrations decreased postoperatively. These findings suggest that tryptophan metabolism and the kynurenine pathway may be altered after shunt vessel ligation. Dogs that developed PANS had lower serum 3OHKYN concentrations on postoperative days 1 and 3 compared with dogs that did not develop PANS, indicating a possible association between postoperative decreases in 3OHKYN and the occurrence of PANS. However, given the limited sample size and observational nature of this study, these results should be interpreted as preliminary findings. Future studies involving larger cohorts and comprehensive analyses of kynurenine pathway metabolites in blood and cerebrospinal fluid will be necessary to further clarify the potential role of tryptophan metabolism in the pathogenesis of PANS and to inform the development of preventive and therapeutic strategies.

## Data Availability

The data presented in this study are included in the article/supplementary material. Further inquiries can be directed to the corresponding author.

## Supplementary Materials

The following supporting information can be downloaded at: https://www.mdpi.com/article/10.3390/vetsci13040308/s1, Table S1: Detailed signalment of each case; Figure S1: Changes in serum CRP concentrations following shunt vessel ligation; Figure S2: Correlation of QA, 3OHKYN and CRP.

## Abbreviations

The following abbreviations are used in this manuscript:

PANS	Postattenuation neurologic signs
cPSS	Congenital portosystemic shunts
QA	Quinolinic acid
3OHKYN	3-hydroxykynurenine
CSF	Cerebrospinal fluid
ELISA	Enzyme-linked immunosorbent assay
