# Supplementary material for: Postoperative Serum Quinolinic Acid and 3-Hydroxykynurenine in Dogs with Congenital Portosystemic Shunt: A Pilot Study of Their Association with Postattenuation Neurologic Signs
Source: Vet Sci. 2026 Mar 24;13(4):308. doi: 10.3390/vetsci13040308 (PMC13120242; doi:10.3390/vetsci13040308)
Supplement: Supplementary file 1 [file vetsci-13-00308-s001.zip › Supplementary_FigureS1.pdf]

Postoperative Serum Quinolinic Acid and 3-Hydroxykynurenine in Dogs with Congenital Portosystemic Shunt: A Pilot Study of Their Association with Postattenuation Neurologic Signs

Shoma MIKAWA, Yuto ISHIMARU, Yasuhiko OKAMURA

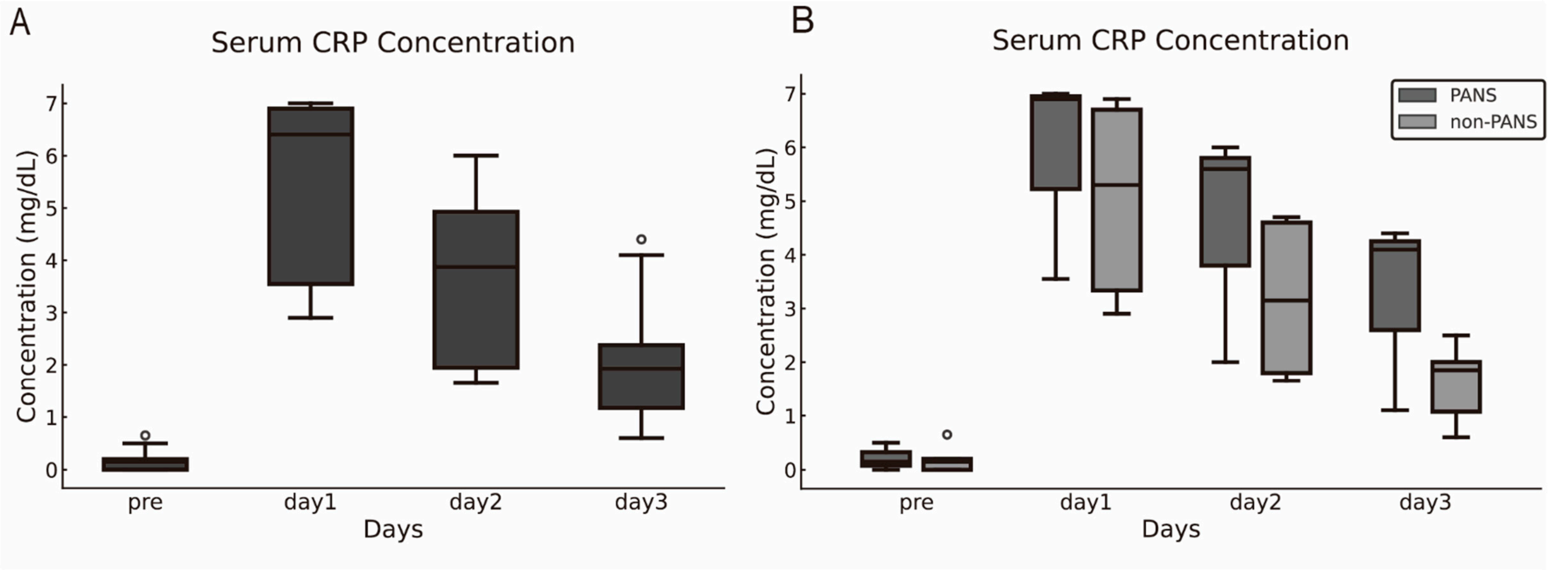

Supplementary Figure S1.

Changes in serum CRP concentrations following shunt vessel ligation in dogs with cPSS. Serum CRP concentrations increased after surgery and tended to decrease over time, but no significant differences were observed between days (A). Similarly, there was no significant difference between the PANS group and the non-PANS group (B).

CRP, C-reactive protein; cPSS, congenital portosystemic shunts; PANS, postattenuation neurologic signs
