# Supplementary material for: Postoperative Serum Quinolinic Acid and 3-Hydroxykynurenine in Dogs with Congenital Portosystemic Shunt: A Pilot Study of Their Association with Postattenuation Neurologic Signs
Source: Vet Sci. 2026 Mar 24;13(4):308. doi: 10.3390/vetsci13040308 (PMC13120242; doi:10.3390/vetsci13040308)
Supplement: Supplementary file 1 [file vetsci-13-00308-s001.zip › Supplementary_FigureS2.pdf]

**Postoperative Serum Quinolinic Acid and 3-Hydroxykynurenine in Dogs with Congenital Portosystemic Shunt: A Pilot Study of Their Association with Postattenuation Neurologic Signs**

Shoma MIKAWA, Yuto ISHIMARU, Yasuhiko OKAMURA

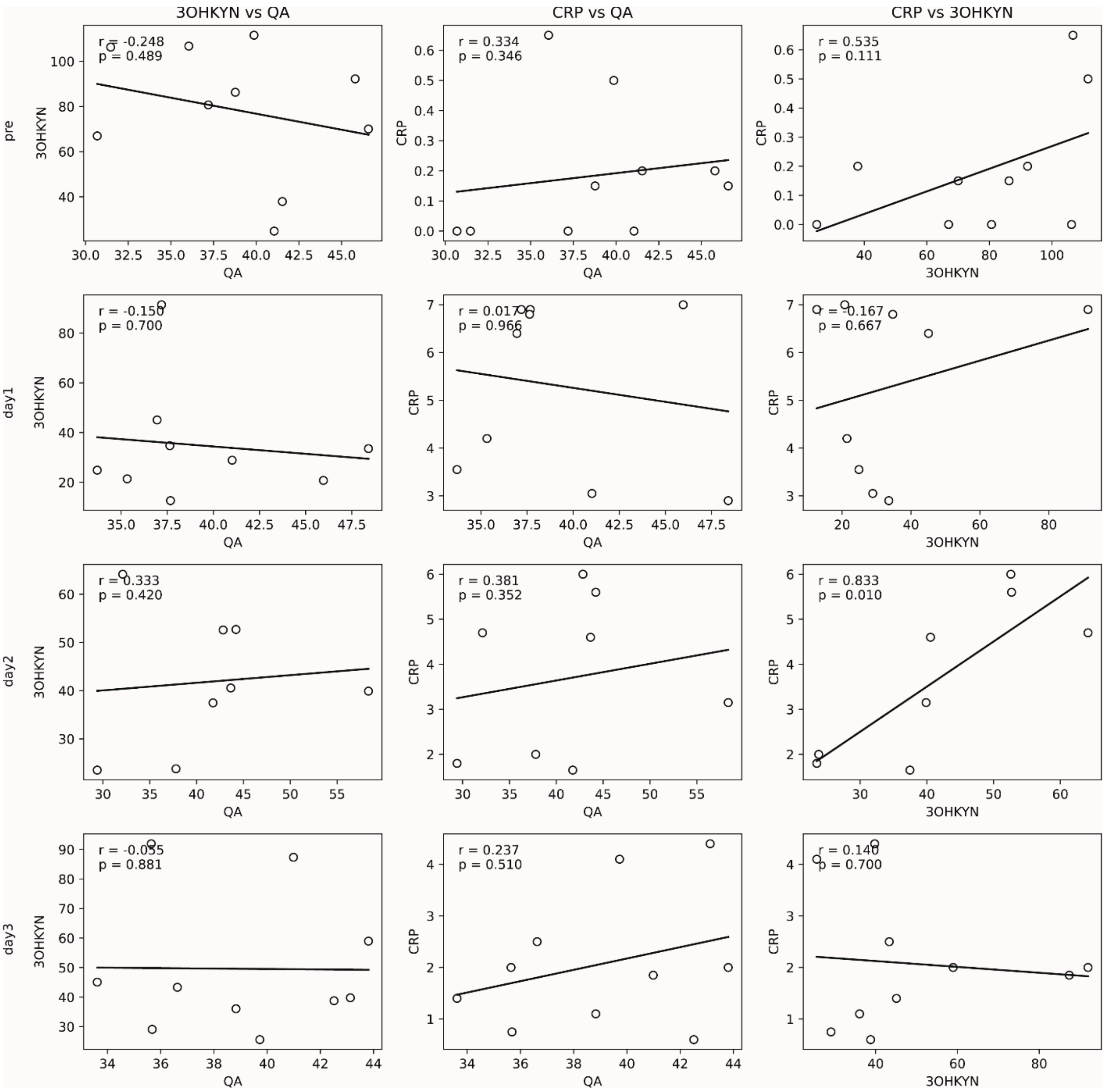


**Supplementary Figure S2.**

Correlation of QA, 3OHKYN and CRP in preoperative, postoperative day 1, postoperative day 2, and postoperative day 3. Only CRP vs. 3OHKYN on postoperative day 2 showed a significant positive correlation.

QA, quinolinic acid; 3OHKYN, 3-hydroxykynurenine; CRP, C-reactive protein
