# Supplementary material for: Postoperative Serum Quinolinic Acid and 3-Hydroxykynurenine in Dogs with Congenital Portosystemic Shunt: A Pilot Study of Their Association with Postattenuation Neurologic Signs
Source: Vet Sci. 2026 Mar 24;13(4):308. doi: 10.3390/vetsci13040308 (PMC13120242; doi:10.3390/vetsci13040308)
Supplement: Supplementary file 1 [file vetsci-13-00308-s001.zip › Supplementary_TableS1.pdf]

Postoperative Serum Quinolinic Acid and 3-Hydroxykynurenine in Dogs with Congenital Portosystemic Shunt: A Pilot Study of Their Association with Postattenuation Neurologic Signs

Shoma MIKAWA, Yuto ISHIMARU, Yasuhiko OKAMURA

Supplementary Table S1. Detailed signalment of each case

Dog	PANS	Breed	Sex	Age (y)	BW (kg)	Shunt morphology	Hepatic encephalopathy	Preoperative Levetiracetam	Therapeutic food	Lactulose	Other Drugs	Procedure	Concurrent treatment	Complications
No. 1	PANS	MIX	male	3.9	5.8	portocaval	−	+	+	+	BCAA, Zinc	Laparotomy	Cystotomy	Urolithiasis
No. 2	PANS	Toy Poodle	male	1.8	3.1	portophrenic	+	+	−	+	BCAA, Zinc	Laparotomy		
No. 3	PANS	Miniature Schnauzer	cast	6.8	7.9	portophrenic	+	−	+	+	BCAA, Zinc, Vit K, Ursodeoxycholic acid, SAMe	Laparotomy	Cholecystectomy	Gallbladder Mucocele
No. 4	non-PANS	Yorkshire Terrier	spay	1.3	2.0	portophrenic	−	−	−	+		Laparotomy		
No. 5	non-PANS	Miniature Schnauzer	male	2.7	8.3	portophrenic	−	+	+	+		Laparoscopic→ Laparotomy	Castration	

No. 6	non-PANS	Shih Tzu	femal	0.7	5.8	portophrenic	−	+	+	+		Laparoscopic	Sterilization	Optic nerve hypoplasia
No. 7	non-PANS	Toy Poodle	cast	1.5	2.8	portophrenic	−	+	+	+		Laparoscopic→ Laparotomy		
No. 8	non-PANS	Toy Poodle	cast	1.5	2.7	portophrenic	−	+	+	+	BCAA, Vit K	Laparoscopic		
No. 9	non-PANS	MIX	cast	2.0	6.2	portocaval	−	+	+	+		Laparotomy		Urolithiasis
No. 10	non-PANS	Chihuahua	spay	2.8	2.5	splenophrenic	−	−	+	+		Laparotomy		

PANS, postattenuation neurologic signs; BW, body weight; BCAA, branched-chain amino acid; SAMe, S-adenosylmethionine
